# Dramaturgical Accounts of Transgender Individuals: Impression Management in the Presentation of Self to Specialist Gender Services

**DOI:** 10.1007/s10508-021-02028-2

**Published:** 2021-11-05

**Authors:** Katrin Lehmann, Michael Rosato, Hugh McKenna, Gerard Leavey

**Affiliations:** 1 Bamford Centre for Mental Health and Wellbeing, Ulster University, Cromore Road, Coleraine, Northern Ireland BT52 1SA UK; 2 Institute of Nursing and Health Research, Ulster University, Newtownabbey, Northern Ireland UK

**Keywords:** Gender dysphoria, Service experience, Impression management, Mental health

## Abstract

Demand for gender dysphoria (GD) treatment has increased markedly over the past decade. Access to gender-affirming treatments is challenging for most people. For dysphoric individuals, much is at stake. Little is known about the specific needs, challenges, and coping strategies of this hard-to-reach group. We examined the experiences of treatment-seeking adolescents and adults using in-depth unstructured interviews with 26 people attending specialist gender services and 14 transgender people not referred to services. Patients with gender dysphoria distrust clinical services and describe considerable anxiety in sustaining their impression management strategies to obtain treatment. An authentic presentation is regarded by some participants, especially non-binary individuals, as inauthentic and emotionally difficult to maintain. Impression management strategies have partial success in accessing services. The presentation of “idealized” selves may result in unmet mental health needs of patients, and the receipt of interventions incongruent with their authentic selves.

## Introduction

### Gender Dysphoria

Gender dysphoria refers to distress experienced due to the incongruence between a person’s inner perception of their gender and an incompatible bodily reality. Symptoms include strong and persisting cross-gender identification and a desire to be of the other gender (American Psychiatric Association, 1994). This can be expressed as distress because of birth gender body parts, or with having to dress or act in ways associated with birth-assigned gender roles. Such distress is unique to each individual and can manifest in different ways (Cooper et al., 2020), with treatment derived from guidance from the World Professional Association for Transgender Health (WPATH) (Coleman et al., 2012). Recent qualitative studies have focused on the experiences of individuals accessing specialist gender services (Goldberg et al., 2019; Lykens et al., 2018; Taylor et al., 2019), with non-binary or gender queer participants highlighting their mixed experience with healthcare professionals—negative interactions based on invalidation, overemphasis or avoidance in respect to gender identity (Goldberg et al., 2019). Similarly, Lykens et al. (2018) noted that non-binary and gender queer participants reported feeling misunderstood by service providers and borrowed a binary identity in order to access treatments, and Taylor et al. (2019) noted how participants described feeling invisible in a binary world. Unfortunately, these challenges are not unique to non-binary or gender queer individuals, with treatment-seeking individuals highlighting the need for both patient-centered care and the agency and power to influence their own treatment decisions (Eyssel et al., 2017). Cruz (2014) highlights stigma and discrimination as factors leading to postponement of access to gender affirming care, while Wilson et al. (2015) stress the importance of addressing racial/ethnic and gender identity disparities in transition-related healthcare.

There is little research on individuals outside specialist gender services, but a recent study (Nieder et al., 2020) exploring the characteristics of individuals not seeking interventions highlighted avoidance of treatments by some due to transition-related suffering (health risks pertaining to hormones and surgical treatments), while others considered such treatments personally unnecessary. Limited knowledge of this diverse population shows the need for more nuanced understanding of—for example—the personal, social and service determinants of help-seeking, and intervention preferences. The stigma, uncertainty and controversy that persists around gender dysphoria (Coleman et al., 2012) and highly oversubscribed services suggests significant help-seeking challenges (Lewis et al., 2017) that require further examination.

Goffman (1959) focused on social interactions by observing, describing and analyzing everyday human interactions in detail. Using a dramaturgical framework, Goffman explored the concept of the individual as actor, playing a role in their social interactions. Impression management implies the effort made by individuals to present themselves in a favorable light (Goffman, 1959). While originally derived for contexts other than the current one, its core concepts can be applied to persons presenting to gender services: for example, the dramaturgical perspective of frontstage (a performance taking place), and backstage where the performer can be more themselves (Goffman, 1959)—with, in this study, frontstage referring to the impression management (performance) by participants attending specialist services (and staff as the audience), and backstage as the social spaces or environments where no performance is necessary. Impression management also incorporates a team element, implying that the showing can be discredited by poor ensemble performance. Here, we explore whether such analysis is useful, and specifically whether such impression management efforts can be, in effect, discredited. Goffman’s dramaturgical framework provides a mechanism to underpin examination of all social interaction—with participants playing out many different roles in relation to the situations we all find ourselves in, each with definite outcomes in mind—in this case a series of interactions between those being assessed (for whom we present evidence) and those who make the assessment, therefore requiring evidence of both gender related distress and stability of presentation. However, we also critique Goffman’s (1959) portrayal of a normative world (Gouldner, 1970), and whether the original conceptualization of impression management is useful in understanding the presentational dynamics within specialist gender services, in relation to (at least) the sustainability of concealing major aspects of self while simultaneously meeting all other pre-requisites of normal social living (Raffel, 2013).

### The GIFTS Study

GIFTS (Gender Identity—Finding and Transforming Services) is a mixed methods project, with findings from an associated prevalence study reported elsewhere (Lehmann et al., 2020). The qualitative study undertaken here examines the experiences of both users of specialist gender services (Knowing Our Identity—Gender Identity Development Service & Brackenburn Clinic) in Northern Ireland (NI), and a small community-derived sample of individuals living with gender-related distress.

## Method

We followed an inductive logic, involving knowledge-building through interview (Ormston et al., 2014). The first author and interviewer (KL) identifies as a cisgender female with a background in nursing and systemic psychotherapy. Elliot (2016) suggests that trans and non-trans persons have very different stakes in trans-related studies and research. In the planning of the research, we were mindful of our outsider position and have benefitted greatly from the involvement as co-investigators of three experts by experience (see below).

### Participants

Participants were initially recruited through the clinical teams in both the specialist gender identity services (separate adolescent and adult services) for NI. These provide assessment and, depending on age, physical interventions to alleviate gender related distress. Hormone blockers and cross-sex hormones can be accessed through local arrangements by adolescents and adults. For those requiring surgical interventions in adulthood, arrangements accessing gender-affirming procedures can be made with specialist surgical centers in England on a case-by-case basis through additional contractual arrangements. Because of stipulations by the ethics committee, concerned with problems of informed consent, individuals with severe cognitive impairment or those experiencing acute psychotic symptoms were excluded from the study. We acknowledge however, that such participants, if included, could have provided valuable insight about the challenges in navigating services to access gender affirming care.

With lengthy waiting lists for specialist gender services, it was important to capture the experiences of individuals not currently attending specialist services, as little is known about the needs, challenges and coping strategies of this hard-to-reach group. It is also likely that those outside specialist gender services are part of a wider group of individuals living with gender incongruence, many of whom may never seek interventions. Participants in the community sample were recruited through information events for adults waiting for specialist services and through partnerships with trans and LGBT-specific support groups and organizations. Some responded directly to posters displayed in community spaces and waiting areas of local LGBT and trans organizations. Others were recruited through “snow-ball” sampling, with persons who had already completed their interviews asking others who might be interested in participation. All participants provided informed consent prior to participation in the interview and completed a short demography form.

Forty participants aged between 15 and 37 years old were interviewed, evenly split between assigned male at birth (AMAB) and assigned female at birth (AFAB) with (16/26) AMAB in the group attending services and (10/14) AFAB in the community sample. Participants in the service sample tended to be older than those in the community sample (mean 34.6 vs. 23 years) (Table 1).

**Table 1:** Participants demographic characteristics

Characteristic	Service sample *n* = 26	Community sample *n* = 14
*Age in years*		
Range	15–66 years	17–54 years
Mean	34.6 years	23 years
*Assigned sex at birth*		
Male	16	4
Female	10	10
*Self-description*		
Male	4	4
Female	8	0
Transman	4	2
Transwoman	6	0
Non-binary	3	7
Other	1	1
*Highest educational level*		
Degree or above	11	3
Intermediate level	9	7
Least well educated	6	4
*Sexual orientation*		
Asexual	5	0
Bisexual	5	3
Heterosexual/Straight	5	1
Gay/homosexual/lesbian	3	2
Gender plays no role	2	0
Pansexual/panromantic	1	3
Unsure/questioning	5	0
Queer	0	3
Other	0	2

### Measures and Procedure

#### Experts-by-Experience

Three “experts-by-experience” advised us throughout the project and helped design the topic guide. Each of the experts had personal experience of gender-related distress and identified as gender diverse. They all had experience of navigating specialist gender services and were active members of trans-community organizations.

#### Gifts Interview Process

The in-depth interview was loosely structured around a topic guide devised in collaboration with the experts-by-experience involved in the overall design of GIFTS, allowing space to explore other issues unique to the individual. Interviews were conducted in participants` homes, spaces provided by voluntary organizations, and in offices in the specialist gender services. Prior to interview all participants provided written informed consent and were free to withdraw consent at any point.

Generally, all participants were asked: (a) “How long have you lived with this (gender-related distress/incongruence)?”; (b) “When did you first notice/become aware?”; (c) “Who (if anyone) did you tell?”; (d) “What was their reaction?”; (e) “Did you seek support or not?”; (f)“Where did you seek support?”; (g) “What influenced your decision to seek/ not seek support?”; (h) “What other factors made an impact on your life at this stage?”; (i) “How was your experience of support offered?”

Towards the end of the interview, participants were invited to imagine an ideal service for individuals who experience gender related distress.

### Analysis

The interviews were audiotaped and professionally transcribed. Checked and amended transcripts were entered into a qualitative software programme (NVivo version 12) for coding and management. Demographic information for participants attending services and those outside services were added to NVivo and linked to each case (numerical participant ID).

Individual interviews were inductively coded, indexed and emergent themes identified. Initially, four transcripts were analyzed and coded independently by the researcher, assisted by experts-by-experience and co-authors. Data analysis was iterative rather than linear and followed the thematic analysis phases described by Braun and Clarke (2006): (1) familiarization with the data; (2) generating initial codes; (3) searching for themes; (4) reviewing themes; and (5) naming and defining themes.

At the initial stage, the researcher carefully read each transcript, focusing on how the interviewee made sense of their experience. The researcher adopted a thematic process best described by Braun and Clarke (2006), coding for the key issues determined by the main questions and topics but included any unanticipated themes and issues.

#### Thematic Analysis

During the first or open coding stage, we identified themes in relation to:

1. Identity management strategies.
2. Interaction and consultation with health professionals along the pathway.
3. Early recognition—how and when they became aware of their trans identity; and
4. Family and social responses—reactions to the disclosure of their identity.

The second (axial) coding was based on the specific research questions. We reviewed the theme of identity management, noting examples of decision-making about presentation of true self and the factors and beliefs on which they were based. The theme of presentation of self to services, combined aspects of identity management and interaction with healthcare professionals. We examined various sub-themes such as decision-making about access to gender services.

The emotional struggles associated with waiting to access services and the anticipation of service access were recurring themes. We explored how this impacted their daily lives and mental health. As sub-themes we also explored the different pathways to specialist gender services, with specific focus on the crucial point of presentation to services. Given the current service model—mental health professionals acting as gatekeepers to physical interventions—we were interested in what impact this might have on how individuals present themselves to clinical staff.

During the third or theoretical coding phase, we developed this presentation to services theme further, finally developing a theme of impression management, which captured behaviors presented by individuals to influence the perceptions of others towards a particular end. At this stage we incorporated the work of Goffman, where impression management may be described as “efforts made by an individual to present themselves in a favorable light” (Goffman, 1959).

To ensure anonymity and to differentiate between participants, contributions are reported using pseudonyms (with age, and reported gender), generated for analysis purposes only and not resembling participant names in any way.

## Results

### Beliefs and Behaviors

#### Gender Dysphoria Must Be Evident

In response to questions about how participants made sense of their trans identity—the majority spoke about the uniqueness of their experiences of gender dysphoria and how this produces social, emotional and physical distress. While one participant did not experience dysphoria related to their physical body, for the majority seeking gender affirming treatments it was crucial that their gender dysphoria should be visible to others. Participants believed that (their displaying of) gender-related distress was crucial to their assessment at gender services and a positive outcome (acceptance for treatment); failure to do so could mean treatments would be withheld. One service user noted that emphasizing the dysphoria was essential to their impression management:

I definitely think a lot of people immediately focus on the dysphoria, I think I may have definitely focused, like it wasn’t that I was lying about it, but I gave it a lot more attention than maybe I needed to.

(Edward, transman).

Earlier (ICD-10) classifications of gender dysphoria and current DSM-5 classifications (Reed et al., 2016) state that overt gender-related distress must be expressed. However, more recent ICD-11 (2019) guidance re-specifies gender dysphoria as a Condition Related to Sexual Health and states that, while incongruent gender-related distress is common, it is now not required for diagnosis (Reed et al., 2016).

Some participants noted through social media or local community events, that the presentations of other trans-identifying individuals varied considerably. This generated conflicting ideas about impression management, team performance, and who has membership (here the team comprises all those trying to access gender affirming treatments). Some participants resented others for attending services at all, considering it unnecessary. One adolescent attending services noted that some people with “zero” dysphoria were “still using the service when there is a big line for people waiting.” This implies perception of a hierarchy of need for interventions, with those wanting to physically transition and access hormonal and surgical interventions regarded as having priority. Those wishing to access interventions may be regarded by others as “less serious” and consequently less valid in their distress. This may function in an informal internal gatekeeping capacity within community groups or among trans-identified individuals, based on competitive rationing and access to scarce resources. Community participants, worried that their dysphoria may not be taken seriously when they eventually attend services, voiced fears that gender dysphoria could be misdiagnosed as a psychiatric disorder. One community-based participant noted:

People can attribute gender dysphoria as a symptom, …and then it might be I'm not trans, I am just a depressed person who has depersonalisation disassociation.

Charly, (non-binary).

Given this conception of the dilemma, specialist staff might not differentiate between gender dysphoria and mental illness—not addressing mental health difficulties as co-existent (and therapeutically separate) with the dysphoria, leading to further invalidation.

#### Gender Identity Must Be Consistent and Stable

Most participants believed their gender identity needed to be consistent and stable and were concerned that inconsistent presentation could jeopardize access to treatment. A few participants were anxious not to show any doubt about their gender identity and ‘to be confident 100% of the time, any question they asked me, yes 100% man.’ This highlights that in frontstage presentation to services participants strove to reassure staff that their gender identity was stable, even if this was not the case—knowing that instability provoked concerns among clinical staff when irreversible treatment was decided. While this pretence served a function for both participants and staff, it unfortunately did not address underlying issues that may have required further therapeutic exploration.

Other participants reported presenting a consistent image of their gender identity: some choosing to wear the same clothing again and again to preserve this consistency.

…if you’re a transwoman buy a skirt that’s your gender identity service skirt, wear it when you go and they won’t ask questions, like for real like.

Blue (non-binary).

One participant waiting to attend adult gender services noted that other individuals who attended had a “…what’s called a GIC (Gender Identity Clinic) outfit…” to ensure a formal continuity in their gender expression. By wearing specific clothes to represent their consistent gender identity frontstage, some participants clearly performed a role—impression management mediated by context (Giddens, 2009). Wearing the same outfits at successive appointments made it easier for the audience (staff) to recognize their character presented. This juxtaposes with their backstage social context, where some participants described dressing differently. The majority of the young adults dressed for comfort outside their clinic appointments. The majority of older participants described being fearful that dressing outside the socially expected norms of masculinity and femininity might in some way invalidate their presentation to services or that clinicians might question their “real life experience.” Some participants required reassurance from the researcher that there was no expectation in relation to how they dressed during interviews. One participant, less concerned about the performance of gender identity, suggested:

I know that I probably don't have really good male mannerisms, but really at this point I don't care and really because I know who I am, okay other people might not realise I'm male, but as long as I know who I am I'm okay.

(James, male)

### Frontstage Impression Management

#### Compliance with Staff at Gender Services

Most participants described the need to be compliant with staff at specialist gender services in order to access treatments. They were very aware of the gatekeeping function that staff held and stories of individuals being denied treatment were known to circulate within the community. Participants described a range of behaviors when presenting frontstage to services, including compliance with clinical staff, and omitting parts of their history (in particular past mental health history) during assessment. Others relayed concerns about the consequences of being truthful during assessment, maybe introducing delays to treatment, or discharge from the service to facilitate other types of treatment, to address, for example, mental health or addiction concerns. Some participants stated that they “lied a bit” and “omitted some things” for fear of being denied services, suggesting that compliance with staff would ease their process towards interventions. Another participant attending services was concerned about “being perfectly honest with them,” such “dishonesty” including denial of psychiatric challenges or concealing fluctuations or possible doubts about their actual gender identity. Participants were aware of others who lied to staff about their histories. This evokes Goffman’s (1959) concept of dramaturgical loyalty, with everyone acting as if having mutually or implicitly agreed to certain obligations or modes of conduct (with non-agreement—for example, by exaggerating distress—regarded as disloyal). One adolescent attending services noted frustration with how such front stage management garnered advantage in relation to the staff, while others, less exaggerated, were possibly disadvantaged.

I know a few people that have and they’re actually smug about it like really pull the wool over their eyes this time—it angers me a wee bit cos there's a lot of people that tell the truth and it’s not good enough.

(Jason, male)

However, being truthful or loyal appeared to disadvantage some people. One participant described the confusion of having to navigate the assessment process—while trying to comply with staff members the participant noted that it “felt like I was in a game that I didn’t know the rules of.”

### Beliefs and Behaviors

#### Staff Have Binary View of Gender

Several participants attending services perceived a services bias in favor of binary-identified individuals, leading some to “try and be more masculine because then it speeds up the process and makes it easier.” This attitude may be based upon beliefs held amongst the community that “there is a rigid sort of binary enforced within the gender identity clinic,” creating potential dilemmas for those identifying as non-binary. Based on feedback from others, presenting as non-binary at the specialist gender services raises the risk of denial of delayal to treatments. By presenting as unambiguously binary, participants believed such access would be easier, though this meant they had to perform a binary gender role, potentially conflicting with a preferred non-binary identity.

### Frontstage Impression Management

#### Present Ultra-Binary

Some participants described how they tried to present in ways they felt were closer to an idealized version of their true gender, including frontstage efforts to present in (binary) ultra-feminine or ultra-masculine ways—to persuade staff at specialist services of their genuine need to transition, and suggesting that some participants believed staff would only accept an outwardly convincing gender presentation. One participant waiting for services, noted that this perceived clinical expectation encouraged a specific type of performance:

…people feel the need to hyper masculinise, or hyper feminize, or just really overstate their gender, perhaps going beyond the level they are comfortable with because they are afraid that they are going to be denied the services they need otherwise.

(Blue, non-binary).

This suggests that non-binary participants seeking to present an idealized binary version of themselves hide or even deny their non-binary identity. Presenting in ultra-binary ways also applied for binary identified participants, and for some this was part of their initial impression management stance. Other binary-identified service users spoke of going “through the stage of being ultra-feminine”—coming to “appointments with fake tan just because I felt I was coming to a gender clinic.” Over time some modified their frontstage presentation from dressing in ultra-binary ways. For some, the perceptions of others were always less important, and they chose not to present in ultra-binary ways.

### Beliefs and Behaviors

#### Mental Health Must Be Perfect

Transgender health care in Northern Ireland has been provided over many years as part of psychiatric services. However, with the recent ICD-11 reclassification from dysphoria to incongruence and shift from psychiatric responses to conditions of sexual health (Reed et al., 2016), the role of mental health professionals may change. One systematic review highlighted that while people seeking gender-affirming care are more at risk of psychiatric disorders, compared to the cisgender population, this lessened following treatment (Dhejne et al., 2016). Vulnerability to mental illness has justified the incorporation of mental health professionals as integral to transgender healthcare. While many participants in the current study describe co-existing mental health difficulties, it is not possible to say if these relate to their gender dysphoria. Nevertheless, it is commonly understood that access to gender affirming interventions is predicated on careful mental health assessment. Many participants describe experiencing mental health challenges, which some believed were based on how they were treated by others. A number of studies have highlighted that transgender people are frequently victims of multiple types of violence, often facing a lifetime of repeated victimization with particularly high risks of sexual violence, a high prevalence of physical abuse, and bullying (Grossman & D’Augelli, 2006; Grossman et al., 2011). One participant attending services, keen to avoid being denied services because of mental health difficulties, reported that: “it’s really bad, um, pretending that you’re okay.”

The WPATH guidance outlines the role of mental health professionals as: (1) facilitating the diagnosis of gender dysphoria; (2) assessing psychiatric co-morbidity (Dhejne et al., 2016) (3) exploring readiness for gender affirming interventions (Coleman et al., 2012); and (4) supporting the trans person through the health pathway (Dhejne et al., 2016). One participant noted that “a lot of people tend to hide trauma and mental illness and learning disorders and things like that because it is a barrier.”

While there are few instances where gender-affirming treatments may not be offered, stable mental health is an important prior condition. This does not mean that individuals cannot access gender-affirming interventions simultaneously with mental health treatment. Participants were aware of this and expressed concerns that, while mental health difficulties were addressed elsewhere, disclosing these at assessment could delay treatment and were therefore concealed. However, such beliefs were not held by all: for example, one person was complementary about the mental health assessment carried out as part of their comprehensive assessment of gender dysphoria.

### Frontstage Impression Management

#### Access Mental Health Support Elsewhere

Participants were asked about their help-seeking behaviors, with the majority indicating that they sought informal support from friends, families and others within the wider trans community. Some described the difficulties they have experienced during a mental health crisis while trying to support others who are also experiencing their own mental health challenges. Screening-based mental health assessment provoked more negative experiences. Some noted that disclosure of mental health challenges resulted in a focus on psychiatric treatment rather than progression towards gender affirming interventions, leading some to access mental health support outside the service. As one stated—they sought “help outside of the clinic so that they would stop pushing back my transition which was feeding into my anxiety which was making me worse.” This underlines the complex navigation for participants between their dysphoria and resolution of mental health challenges. For example, it was suggested that others had been penalized for disclosing self-harm:

Like they’d been kicked out of GIC just maybe if there was previous self-harm as far as I was aware. Not sure if it was happening at the time but it was—like—a lot of things—they rather kind of had to keep that little bit in and deal with it as a secondary issue.

(Brenda, transwoman).

### Backstage Outcomes of Impression Management

#### Frustration Related to Impression Management

Many participants shared their frustration at having to describe to clinicians why they required gender affirming treatments. For some participants, the idea that anyone apart from themselves could possibly be able to understand or even assess their internal sense of themselves seemed inconceivable. Impression management was problematic for some participants—echoing Garfinkel’s (1967) critique of Goffman (1959), which argues for the impossibility of sustained concealment of salient aspects of gender identity. One participant attending services described the frustration of having to prove her transgender status while engaging in impression management beyond a level she might have chosen:

I found the frustration coming here was having to prove myself to be transgender to get this referral and it’s like you’re not really being your authentic self because you’re dressing up more you’re looking more feminine, even to the point where you might have like gone long enough for in your everyday life but you’re just trying to prove here that you are because you want to get it so badly. And I think it’s a real shame because you’re not being honest with yourself.

(Sarah, transwoman).

Thus, impression management undermined a sense of authenticity related to gender identity. Some people reduced their reliance on this over time, reporting increased comfort in dressing in a way that matched their preferred gender identity.

#### Exhaustion Related to Impression Management

Participants reported negative effects of impression management, including mental and physical exhaustion. Attending appointments at the specialist gender services was particularly tiring for some participants who found the appointment process overwhelming. In GIFTS, many participants portrayed multiple identities—for example, identifying as trans (binary or non-binary), disabled or having various mental health diagnoses. Consequently, the felt need for impression management appeared to increase their exhaustion and frustration.

#### Coping Without Mental Health Support

Successful impression management for some meant they could hide their mental health difficulties. While this reassured staff and allowed them to access interventions, they were left without access to mental health support. One noted:

I was feeling really, really low, you know, in very, very bad, like the first time in years, like considering self-harm, like really self-destructive behavior, super depressed, struggling to sleep, just nightmares, flashbacks, the works. I really should have been able to say to my therapist, I’m having a bad time, can we talk about this? Was I going to do it? No. No way.

(Edward, transman)

The decision to essentially withhold information about his deteriorating mental health was based on hearing about other peoples’ experience of gender affirming treatments being delayed or stopped by clinical staff. Edward “didn’t know what the repercussions [of disclosing his poor mental health] could be and wasn’t going to risk it.”

After successful engagement with impression management, some felt unable to subsequently modify their behavior for fear of gender affirming treatments being taken away. Some, who subsequently adjusted their impression management behavior, experienced delays to gender affirming treatments, reporting benefits of physical transition but struggling also with imposed delays. One participant attending services noticed an overall improvement since transitioning but was annoyed that physical interventions were only offered when staff were unaware of his mental health struggles. He reported that staff should have taken his anxiety symptoms as an indication of help-seeking:

I need help rather than let us put this [transition] on hold while we deal with this.

(Patsy, male).

## Discussion

This study used a clinical and community sample to examine the experiences of individuals living with gender dysphoria. We examine how participants conveyed their gender dysphoria (impression management) to the clinical staff deciding on their treatment options. Foucault’s concept of the gaze outlines a way of looking at or comprehending the world (Foucault, 1973): for example, where the medical gaze is an act of viewing people in relation to specifically medical knowledges and sensitivities gathered by the one who gazes, and how this predominates as an extension of an essentially reductive and unidirectional power relationship (perceived as a decision-making process by the person doing the presenting). In this scenario, clinical staff confirm or reject the authenticity of participants by focusing on specific aspects of a presentation to the service. It could be speculated that clinical staff had greater experience with binary presentations and were less familiar with non-binary presentations. Goffman (1959) described this as dramaturgical discipline—essential for maintaining overall performance.

Using Goffman’s (1959) dramaturgical conceptualization, we highlighted that participants present frontstage with service staff forming the audience. In this study, impression management strategies were motivated by beliefs held about staff in specialist gender services: that they (1) held binary views about gender identity expression; (2) demanded perfect mental health; and (3) expected gender dysphoria and a consistent gender identity. To meet these perceived expectations participants: (1) presented in ultra-binary ways even if this was inconsistent with their gender identity; (2) denied any mental health difficulties and tried to access support elsewhere; and (3) were compliant with staff while omitting parts of their histories. Using these strategies participants could successfully access gender affirming treatments, while some non-binary participants experienced delays, confirming widely held beliefs that presenting as binary ensures faster access to interventions. Consequently, participants reported exhaustion and, paradoxically, further dysphoria, simply because their managing of impressions required a distortion of self. Such denial of mental health challenges to avoid delay in accessing treatments increased the risk of suicidal ideation.

Goffman's (1959) theorization of the social world as a stage in which social interactions are performed to describe social interactions have been criticized (Giddens, 2009), and Goffman himself acknowledged some obvious shortcomings. The idea of the social world as a dramaturgical setting was difficult to apply to the current social milieu, raising several issues of its applicability in this study. Based on Goffman (1959), participants should be expected to mutually define an implicit team strategy to promote unified presentation strategies as part of a collective impression management. It was clear from the interview data that there were differences between participants seeking gender-affirming treatments. Because there were no such agreed standards amongst participants this created difficulties in relation to dramaturgical loyalty and many participants were not consistent in their impression management in their impression management, some modifying their presentation from ultra-binary to a style congruent with their personal identity. Others did not adhere to perceived norms of masculinity and femininity and trusted their own sense of self, irrespective of outward appearance.

**Fig. 1:**
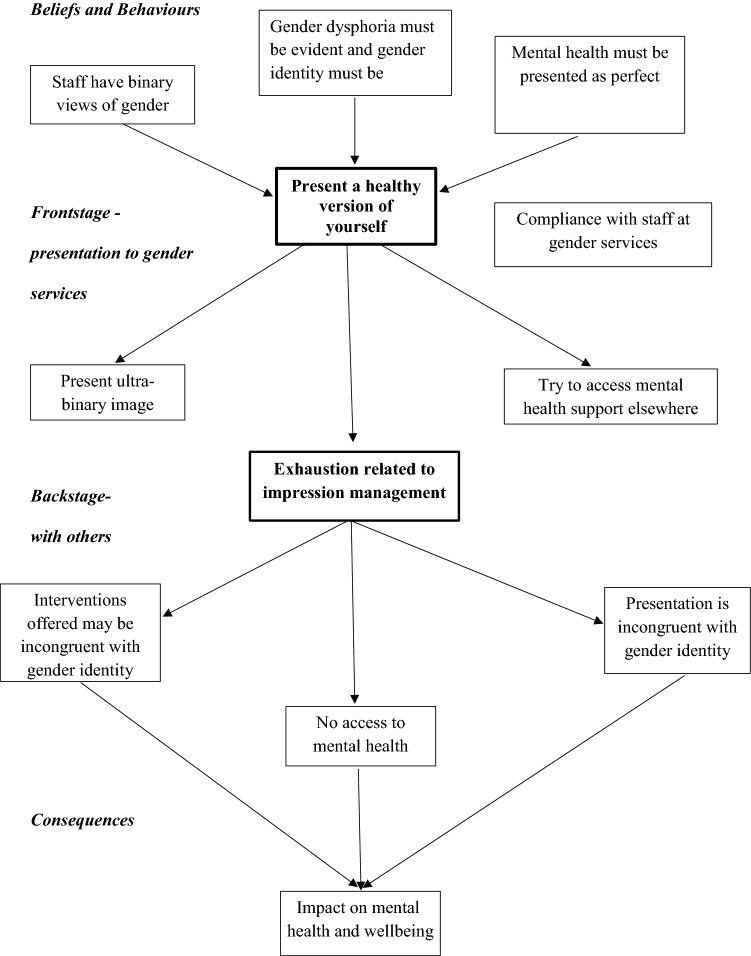
Impression management

Gouldner (1970) argued that impression management portrays a normative world in which appearances and not underlying essences or morality are promoted, thereby echoing Rousseau and his view of appearance as a mask of insincerity and a barrier separating individuals. In this scenario, gender dysphoric participants may be seen to consciously and strategically use impression management in the hope of accessing desired interventions, with the suggestion that participants can dupe staff and that their lived experience is little more than an act, invalidating any lived reality of study participants. A study of non-binary or gender queer adults by Lykens et al. (2018) suggests that individuals borrow a binary identity label in response to a perceived bias from clinicians. In reality, clinicians recognize that individuals are usually very distressed when they seek services and may challenge any discrepancy between an individual’s presentation to services and their authentic self. This process requires trust and reassurance from services that this will not lead to exclusion from gender affirming treatments. Some participants in this study appeared to be influenced by the negative experiences of others and persistent myths in the community, while others found it very difficult to speak about positive experiences in the service for fear of exclusion from the community (Fig. 1).

It is important to note that Goffman’s (1959) view of interaction within the social world may neglect the issue of power. In these scenarios, although participants use impression management with gatekeeper-staff to gain access to scarce gender affirming treatments, these practices do little to address the power balance between participants and clinicians. Other studies highlight the importance of person-centered care, involving individuals in decision-making about gender affirming treatments (Eyssel et al., 2017). Moreover, clinical staff work to clinical guidelines that require evidence of gender-related distress and a stable presentation, creating a bind for both service users and clinicians. This application of Goffman`s ideas has provided a framework to explore impression management in individuals seeking gender affirming treatment. It highlights the shortcomings of typical histories of distress, providing an opportunity to widen the debate about different narratives of gender related distress, which in turn will encourage individuals to present themselves in a more authentic way to services.

Nieder et al. (2020) indicate scant academic literature in relation to people with gender dysphoria not seeking gender-affirming treatments. Decisions related to accessing gender affirming treatments are limited by financial constraints in countries like the United States (Crewe, 2015) and may therefore be transient states (Nieder et al., 2020) depending on access to financial means. There is growing understanding that some individuals do not transition out of lack of necessity as they do not experience distress related to their physical bodies (Karlan, 2016). Wider societal acceptance and a more expansive understanding of gender would allow new ways of defining spaces of identification for individuals Pardo (2019).

### Conclusion

This study explored the lived experience of gender related distress. A unique aspect of this study was the involvement of experts-by-experience, ensuring the study was culturally appropriate and sensitive to topics important to individuals living with gender dysphoria. Clinicians offering access to physical interventions required for gender affirmation must be certain that individuals have been appropriately assessed and can provide informed consent prior to (often irreversible and expensive) physical treatments. Such decisions informing access can be influenced by how treatment-seeking individuals present at assessment. Typical presentations of gender dysphoria (often extensively described in the literature) can reassure staff and promote a satisfactory physical intervention outcome. Co-existing mental health difficulties may persist even after physical interventions. Individuals accessing specialist services are aware of the assessment processes and may feel it necessary, due to perceived bias from clinical staff, to perturb their presentation sufficiently to guarantee their desired treatment. Therefore, while impression management may appear superficial it is therefore undertaken to achieve true gender identity. Based on the findings of this study, it is clear that trust has to be built between clinical staff and those wishing to access gender affirming care. The perceived power imbalance between people accessing and those providing gender affirming care has created an unhelpful dichotomy. Trust requires transparency about decision making, detailed explanations and rationales for delays in treatment access and publicly accessible information about pathways to care. These should be created in partnership with individuals and organizations representing those with lived experience of gender related distress, with particular emphasis on treatment access for non-binary and gender queer individuals.

### Limitations

All GIFTS interviews were completed face-to-face, with participants visible to the single researcher (KL). While most had already socially transitioned and were therefore visible, the trans identity of some was not known to others, rendering their identity essentially invisible. For those attending specialist services, this was less problematic. Participants expressed fears about how they presented themselves to the researcher, but this was generally offset by the support for the study within the transgender community. Some also worried that how they presented during the interview may be reported back to the clinical service and that this might impact on the validity of their transition. While participants were reassured during the interview, phone interviews may have been easier for some participants. Because this study excluded those experiencing cognitive impairment or psychotic symptoms, their experience of navigating healthcare services has not been captured in this study.

## Data Availability

Due to the sensitive nature of this study, ethical agreements do not allow public sharing of the research data. We are happy to facilitate any queries or questions related to the research as per ethical agreements.
